# Internationalization Issues in Chinese Firms: One Belt, One Road-Based Perspective

**DOI:** 10.3389/fpsyg.2022.881155

**Published:** 2022-06-01

**Authors:** Xia Wu

**Affiliations:** School of Historical Culture and Tourism Management, Liaocheng University, Liaocheng, China

**Keywords:** employee brand-based equity, internationalization issues, HRM practices, brand knowledge dissemination, organizational identity

## Abstract

The underlying aim of this study was to investigate the impact of human resource management (HRM) practices, organizational identity, and brand leadership on employee brand-based equity through the mediatory role of brand knowledge dissemination. A questionnaire was adopted to obtain data from 421 employees working in the construction sector of China. The SmartPLS software was used to analyze the data with the help of a structural equation modeling (SEM) technique. The results revealed that HRM practices and organizational identity had a positive and significant relationship with employee brand-based equity, while brand leadership had no direct impact on employee brand-based equity. The results also revealed that brand knowledge dissemination mediated the relationship between independent variables (HRM practices, organizational identity, and brand leadership) and dependent variable (employee brand-based equity). Theoretically, this paper made a valuable contribution by examining the impact of HRM practices, organizational identity, and brand leadership on employee brand-based equity. In terms of practical implications, this study would obviously help the organizations to improve their employee brand-based equity through HRM practices and organizational identity.

## Introduction

Once China’s Go Global strategy was implemented, Chinese companies have been increasingly interested in internationalization, whether it may be through direct investment or cross-border acquisitions and mergers (Buckley et al., 2007; Wei, 2010). Therefore, China’s outbound foreign investment has been gradually increasing. Likewise, China, which was traditionally linked to enormous private investment outflows, has emerged as a significant global investor (Liu et al., 2005). Only the United States surpassed the yearly investment level of 183 billion dollars in 2016 (Haasis and Liefner, 2019). The enormous globalization of Chinese enterprises has already been extensively monitored and actively discussed among researchers (Blomkvist and Drogendijk, 2013).

The origins and types of Chinese enterprises’ internationalization, and the ramifications for China’s economic catch-up process and the global economy, are the central focus of this argument (Lattemann and Alon, 2015). Researchers have emphasized the major involvement of the government and state-owned businesses in the internationalization of Chinese firms, often in opposition to the internationalization trends of developed corporations and nations (Yang and Stoltenberg, 2014; Li et al., 2016). Scholars have also looked at how Chinese enterprises keep up to the west rivals despite a lack of firm-specific strengths, as well as the influence of Chinese firms’ internationalization on their economic condition (Peng, 2012; Clegg et al., 2016). Moreover, empirical evidence on Chinese enterprises’ internationalization is still in its infancy and fragmented (Hao et al., 2020b).

Deng made one attempt to resolve the scattered nature of research on Chinese enterprises’ internationalization. Deng evaluated the academic literature on the research subject from 1990 to 2010, grouping the surveys into three key groups (origins, procedures, and consequences of Chinese enterprises’ internationalization) (Deng, 2012). One belt, one road initiative was initiated during early years of the second decade of 2000 for economic integration (Shahriar, 2019). One of the goals with this project is to increase China’s commercial viability by spending on infrastructure in Africa, Europe, and Asia. One belt, one road is the greatest infrastructural plan ever implemented by a particular country. China’s ambition is to develop the world’s biggest campaign to showcase business and financial links with the entire world under its auspices (Pandey, 2018). China is allowing its corporations, largely attributed businesses, to transfer their knowledge in these sectors after modernizing the place with strong train lines, highways, and electricity.

China’s “going-out” policy has evolved through time, with each iteration aiming to further China’s trade integration (Das, 2017). This could be possible with the identification of organizational challenges of Chinese firms linked to internationalization challenges. In these circumstances, human resource management (HRM) is the most significant and sensitive of all management areas in the local and international environment (Zhang, 2022). The most popular method of starting a new industry—acquisitions and mergers—necessitates more effort and management expertise in disseminating and integrating a strategy of the company, values, and practices, which is solely the domain of HRM. HRM has evolved along with firms’ internationalization policies, such as international employment and talent practices which are linked to the whole company’s strategy (Lee et al., 2021).

HRM is unquestionably helpful in the internationalization process, but it is rarely invited to strategic meetings. Because of a variety of issues, including a failure to demonstrate the required competency (Subramony et al., 2021). Organizations’ desire to increase productivity through HRM practices has brought up the question of what capabilities, expertise, and knowledge HR managers require applying such practices and assisting the internationalization process (Cascio and Boudreau, 2016). Organizational identity is a psychological paradigm that refers to what employees consider to be fundamental, distinctive, and long-lasting about their company (Albert and Whetten, 1985). Transforming a firm’s identity necessitates modifications to organizational members’ internal psychological structures and attitudes, which can lead to resistance and the inability to execute change (Nag et al., 2007).

Whereas the majority of this study has focused on existing organizations, altering the organizational identity of new ventures is as difficult and can take longer than the age of the venture at the time the transition occurs (Tripsas, 2009; Snihur and Clarysse, 2022). Such results emphasize intriguing questions about the impact of organizational identity all through internationalization, as well as the interaction between organizational members’ cognition and the ongoing change implied by pivoting in nascent markets, which is frequently depicted by academics and practitioners as seemingly smooth transitions (Blank, 2013; Marx and Hsu, 2015). Customers’ decision-making procedure to acquire goods and services is influenced significantly by brand leadership. Authenticity, affordability, innovativeness, and popularity are four important elements of brand leadership as viewed by customers.

Firstly, quality is described as “consumers’ assessment of a product’s relative superiority in the marketplace.” Secondly, “consumers’ appraisal of a product’s relative financial value based on what they provide and get” is what value refers to (Miller and Mills, 2012; Chang and Ko, 2014). Thirdly, consumers’ perceptions of a brand’s relative capacity to be open to creative ideas and work on new solutions are described as “consumers’ perceptions of a brand’s relative capability to be open to innovative ideas and work on new solutions.” Finally, “consumers’ sense of a brand’s relative popularity as represented by brand awareness and consumption” is what popularity refers to. Consumers also want brand leadership to have a clear vision and to stay current (Akbar et al., 2021; Khamwon and Sorataworn, 2021).

Internal branding, according to academics, may be utilized to maintain uniform staff behaviors and attitudes (Burmann and Zeplin, 2005). Employer branding contributes to employee behaviors and attitudes by delivering relevant and meaningful brand information. Furthermore, such psychological and cognitive shifts mean that workers transmit the brand value to customers as planned (Avotra et al., 2021; Yingfei et al., 2021). As a result, it is critical to disseminate brand knowledge inside the corporate branding. Workers, for instance, are unable to convey an organization’s brand identity to consumers lacking brand-related knowledge. Moreover, few stated that “workers may feel directionless, grappling with understanding where, when, or to whom to devote their energy” in the lack of brand awareness (Zulkepli et al., 2015).

Employee-based brand equity (EBBE) relates to the perception of additional value that workers obtain as a consequence of brand-building activities (Helm et al., 2016). Employees’ internalization of the company’s values is a crucial element in internal branding, as continuous execution of the brand promise to consumers is improbable without it. Internal branding success depends on internal stakeholders’ alignment with the company’s values and how this interpretation translates to brand- and/or customer-related behaviors (Du Preez et al., 2017). Consumers’ experiences with the brand promise will remain ineffective without employees’ orientation with the brand value (Boukis and Christodoulides, 2020). It was necessary to develop EBBE and evaluation of determinants of EBBE in the context of internationalization of Chinese firms while thinking about economic developing in one belt, one road perspective.

In the perspective of one belt, one road, there was a need to integrate the concept of internationalization of Chinese firms and associated challenges in terms of developing internal brand equity. Based on this gap in previous studies suggested by García Álvarez de Perea et al. (2019), the following research tried to signify the internationalization challenges associated with one belt, one road initiative at the organizational level of Chinese firms. Moreover, the mediating role of brand knowledge dissemination toward developing employer-based brand equity was not studied before, and to overcome this gap, it was suggested by Zulkepli et al. (2015) to evaluate the mediating role of brand knowledge dissemination which could lead to the development of EBBE. Therefore, this research was carried out focusing the impacts of HRM practices, organizational identity, and brand leadership on EBBE through mediation of brand knowledge dissemination.

## Theoretical Underpinning and Hypothesis Development

According to resource-based theory (RBT) (Zica et al., 2016), good business performance is dependent on a unique set of strategic resources that the firm must possess and successfully utilize (Mikalef and Gupta, 2021). The financial, technical, human, and organizational resources that a company employs to design, manufacture, and provide services or goods to its clients are all effectiveness of organizational resources (Agyabeng-Mensah and Tang, 2021). To meet the RBT’s standards, businesses must have high-quality human resources available to meet client expectations and remain competitive in the marketplace (Islam et al., 2021). Employees are a strategic tool for companies to create and build relations, particularly knowledge-based strategic resources that are unique to the company in which they work (Chowdhury et al., 2022). As a result, an organization’s leadership, in collaboration with human resource (HR) experts, must utilize HRs for intra-organizational and cross-functional interaction in the most effective way to achieve organizational goals (Germain and McGuire, 2022).

This theory provided basis for analyzing the impact of HRM and brand leadership for internalization of organizations. Identification-based relationships, on the contrary, are found here in social identity theory, which explains employee relationships as a match among personal and corporate identities (Erkmen, 2018). The social identity theory (SIT) is the foundation of this research. The SIT was used in a variety of settings, including psychology of consumers, information dissemination, and the connection between sports franchises and their supporters (Dimofte et al., 2014; Mckinley et al., 2014; Ambrose and Schnitzlein, 2017). The SIT is a core theory in cognitive science that has been used to explain group psychology, interacting, and social perspectives. It was proposed by Tajfel and Turner (2004).

The component of one’s self-concept that stems out from social group or groups to which someone belongs, as well as the significance and psychological value linked to affiliation to an organization, is referred to as social identity. It is the aspect of self-identity that is mostly generated from belonging to a group (Tajfel and Turner, 2004). People tend to associate and link themselves to diverse social groups as a way of selecting self-identity and a feeling of belonging. As per this theory, employees find the importance and significance of organizational identity. As a result, humans form a sense of social identity regarding the social characteristics of the groups to which they belong, such as race, ethnicity, gender, and political party (Chan, 2016). Therefore, this theory provided basis for analyzing the impact of organizational identity on EBBE.

### Human Resource Management Practices Significantly Impact the Employee Brand-Based Equity

Human resource management practices describe the management methods that enable firms to obtain valuable and outstanding information, as well as impact creative activity and greater performance (Cao et al., 2021). HRM impacts workers’ job-related attitudes, talents, and behaviors in order to achieve an organization’s goals, and it plays a crucial role in creating an atmosphere that is conducive to knowledge management and innovation (Wen et al., 2021). Because knowledge and innovation have origins in human psychology, numerous previous studies have recommended adopting HRM strategies to better encourage the development and dissemination of knowledge for improved organizational innovation performance (Than et al., 2021). The goal of knowledge-based HRM (KHRM) practices is to improve knowledge processes inside a business (Noopur and Dhar, 2020).

Through particular recruiting and selection, training and career development, performance assessment, and compensation procedures, HRM techniques attempt to improve the flow of knowledge—knowledge acquisition, absorption, transformation, and exchange activities in the business (Roundy and Burke-Smalley, 2021). HRM techniques enhance an organization’s human capital foundation while focusing on less apparent value-generating elements such as engagement and knowledge embedded in scalar networks (Ahmad et al., 2021). Individual employees in a business can network with coworkers to establish interpersonal ties through HRM practices (Abugre and Acquaah, 2022).

Employees’ creative behavior and overall organizational innovation in the social enterprise are encouraged by HRM methods based on the opportunity–motivation–ability approach (Azeem et al., 2021). As HMR improves knowledge flows in the business, this research advises reinventing and restructuring traditional HRM into HRM to enable coworkers to collaborate in knowledge production and sharing activities to encourage employee creativity (Ahmad et al., 2021). The author suggests that an HRM practice is a collection of carefully selected HRM practices targeted at improving organizational knowledge, influencing human capital, co-creating relevant work experience, and employee brand-based equity for improved creative performance inside the business. Therefore, the author proposed the following hypothesis:

H1: *HRM practices significantly impact the employee brand-based equity.*

### Association of Organizational Identity With Employee-Based Brand Equity

Organizational identity emerges through complex, interactive, and reciprocal relationships among administrators, organizational members, and other stakeholders (Cornelissen et al., 2021). The core, unique, and enduring aspects of an organization are characterized as organizational identity (Albert and Whetten, 1985). Researchers saw this prominence as being rooted in the organization’s basic characteristics and differentiation from its competing companies: “organizations preserve identity through engagement with other organizations through a system of cross comparison over time.” Eventually, they understood that these lasting features were rooted in the organization’s long-term stability. Researchers in the subject of organizational identity find it challenging to extend the notion to businesses (Bala et al., 2021).

This sparked several disputes within the organizational identity community, and organizational identity was eventually established from pragmatic, sociological constructionist, postmodern, and psychological viewpoints. In actuality, organizational identity is not mindlessly followed; instead, organizational identity is defined by the meanings that members of an organization ascribe to it. As a result, researchers have begun to study organizational identity from a social constructionist viewpoint, which focuses on an organization’s members’ cognitive capacities in grasping “who we are” as a group (He and Brown, 2013). Organizations, on the contrary, do not operate in a vacuum, and their actions have an impact on society. Members of an organization also perceive organizational identity to relate to “how others see us” in this way (Mujib, 2017).

Managers, who give supportive leadership that fosters staff brand-building activities, benefit firms and favorably influence customers’ brand impression (Xie et al., 2016). An employee’s identity with a company is influenced by his or her work placement, which in turn impacts his or her conduct when communicating with customers. In addition, De Chernatony et al. (2006) asserted that employees are informed about brand values through unsubtle official communications, including cascading effect through one level of workers to another, senior leadership engagement and management, and human resource operations. Employee brand internalization is influenced by how the organization is represented, understood, and conveyed inwardly (Lane Keller, 1999).

Similarly, corporate identity has a significant impact on employee brand internalization. It is clear that executive brand identity has a favorable impact on staff brand internalization (Boukis and Christodoulides, 2020). Transforming a firm’s identity in internationalization scenario necessitates modifications to organizational members’ internal psychological structures and attitudes, which can lead to resistance and the inability to execute change (Nag et al., 2007). The impact of organizational identity on EBBE was reported by Liu et al. (2020), indicating that brand identity among employees directs the positive outcomes of EBBE. Based on this analogy, the author developed the following hypothesis:

H2: *Organizational identity has a significant relationship with employee brand-based equity.*

### Brand Leadership Positively Influences Employee Brand-Based Equity

A brand, according to the American Marketing Association, is “a name, word, design, image, or any other element that distinguishes one seller’s item or service from most of the other sellers (Ngoc and Tien, 2021).” Managers may use brands to create points of difference and long-term competitive advantages by reflecting a product’s economic and functional attributes, as well as important intangible associations such as competence and reliability (Rambocas and Narsingh, 2022). Three significant factors for establishing a meaningful brand are integrated into the brand strategic framework (Thomson et al., 2022).

First, the brand building takes into account both quantitative and qualitative measures such as a company’s reputation, its countries of origin, product characteristics, quality associations, and perceived reliability (Bairrada et al., 2021). Second, brand occurs when a company uses consistent imagery and delivery to reinforce a brand’s meaning and context, which necessitates supportive organizational structures and procedures (Buhalis and Park, 2021). As a result, marketers must use a “bottom–up and top–down strategy to brand creation” to guarantee that employees are actively involved in the process (Van Nguyen et al., 2021). Third, the framework implies that marketers that are unable to engage in standardized branding programs to address the individual demands of their consumer segments must be able to adjust branding programs across countries and client segments. Therefore, the authors proposed the following hypothesis:

H3: *Brand leadership positively influences employee brand-based equity.*

### Mediating Relationship of Brand Knowledge Dissemination

Employee corporate brand experience is defined as every interaction workers have with an employer identity, including a variety of brand touchpoints (Saleem and Hawkins, 2021). And brand knowledge is composed of a particular brand in the memory that is related to a range of connections (Han et al., 2021). The way brand networks are organized in one’s memory affects how brand information is remembered, which in turn affects an individual’s behavior and brand-related behaviors (Farzin et al., 2021). While Keller (1993, 1998) focuses on the consumer, brand awareness is also important for employees (Carlini and Grace, 2021). That really is, brand understanding is the key to employees’ knowing how to execute the brand promise (Boukis et al., 2021).

Employees that lack brand expertise are unable to behave in the manner required by the business or make brand-related decisions, which is compatible with the customer viewpoint (Peng et al., 2021). In the field of branding and marketing management, the concept of EBBE has received a lot of attention. Any company’s brand is one of its most significant intangible assets. Brand equity may be used to analyze the impact of previous marketing activities, evaluate the brand’s current positioning, and forecast the future success. The differential influence of brand awareness on employee response to the workplace is known as EBBE.

Internal branding may be utilized to maintain uniform staff behaviors and attitudes (Burmann and Zeplin, 2005). Employer branding contributes to employee behaviors and attitudes by delivering relevant and meaningful brand information. Furthermore, such psychological and cognitive shifts mean that workers transmit the brand value to customers as planned (Hao et al., 2020a). As a result, it is critical to disseminate brand knowledge inside the corporate branding. Workers, for instance, are unable to convey an organization’s brand identity to consumers lacking brand-related knowledge. Moreover, few stated that “workers may feel directionless, grappling with understanding where, when, or to whom to devote their energy” in the lack of brand awareness (Zulkepli et al., 2015). Keeping in view the significance of brand knowledge dissemination as mediator, the author proposed the following hypotheses:

H4: *Brand knowledge dissemination mediates the relationship between HRM practices and EBBE.*
H5: *Brand knowledge dissemination mediates the relationship between organizational identity and EBBE.*
H6: *Brand knowledge dissemination mediates the relationship between brand leadership and EBBE.*

## Methodology

This study utilized the quantitative research design for the validation of the hypothesis for the present study. The hypotheses of the study helped to examine the effect of predictors on outcomes. This research design helped to eliminate any biases. The data for this study were collected through a self-administered survey. The rationality of data was made sure by making the items of each clear and short. The target population was the employees working in the construction sector. The current study used a convenience sampling technique in order to sample from the target population. This technique is a cost-effective and effective method to acquire data from the respondents (Nawaz et al., 2019). Initially, 500 questionnaires were distributed and the author received 421 responses. The unit of analysis was the employees of the construction sector in China.

### Statistical Tool

SmartPLS 3 software was used in this study for the purpose of data analysis. The technique used in our study is structural equation modeling (SEM). Partial least square is extensively used in management and social sciences as it is a variance-based SEM technique (Nawaz et al., 2020). Moreover, PLS-SEM is a causal modeling approach and its aim is to magnify the explained variance of latent dependent constructs. Researchers view PLS-SEM as “silver bullet” conducive to deal with empirical findings with small sample size (Hair et al., 2011). SmartPLS has a user-friendly interface and contains advanced features (Garson, 2016). Furthermore, the technique used in SmartPLS is finest to serve a research having complex equations (Xiaolong et al., 2021; Nawaz et al., 2022). To calculate the values of beta, reliability, and standard error precisely, this study follows the recommendations of Wong (2013) and ensures that all those indicators are part of their respective latent variables having outer loadings of 0.7 in the reflective outer model evaluation.

### Measurement

A five-point Likert scale was used to obtain data for each item of the variable under study. The reliability of each variable using Cronbach’s alpha should be more than 0.7 (Sarstedt et al., 2017).

#### Human Resource Management Practices

Human resource management practices were measured by using the scale of Aurand et al. (2005) that comprises five items. The Cronbach alpha for this variable is α = 0.927; therefore, this scale is reliable.

#### Organizational Identity

Organizational identity was measured by using items of Hanson and Haridakis (2008) that consist of 17 items. The Cronbach alpha for this variable is (α = 0.920); therefore, this scale is reliable.

#### Brand Leadership

The present study utilized the scale of Morhart et al. (2009) that adopted the items from the multifactor leadership questionnaire from 5X (Avolio et al., 2004) to measure the brand leadership. The Cronbach alpha for this variable is α = 0.924; therefore, this scale is reliable.

#### Brand Knowledge Dissemination

Brand knowledge dissemination was measured by using scale of Foreman and Money (1995) that is comprised of five items. The Cronbach alpha for this variable is α = 0.894; therefore, this scale is reliable.

#### Employee Brand-Based Equity

To measure employee brand-based equity, the scale of Schivinski and Dabrowski (2014) was used consisting of 19 items. The Cronbach alpha for this variable is α = 0.920; therefore, this scale is reliable.

### Demographic Details

The demographic details of the respondents who have participated in the study are discussed below. The total participants of the study were 421, and out of them, 270 were males and 151 were females. The employees between the age bracket 20 and 30 years were 29%, between 30 and 40 years were 43%, between 41 and 50 years were 20%, and above the age of 50 years were 8%. Furthermore, the employees with an organizational tenure of less than a year were 46.20%, the employees with an organizational tenure of between 1 and 3 years were 40.63%, the employees with an organizational tenure of between 4 and 6 years were 7.98%, while the employees with an organizational tenure of more than 6 years were 5.19%.

### Common Method Bias

This study used a single Harman’s factor test to analyze common method variance in order to check for common method bias in the data (CMV). SPSS 21 was utilized in this study to perform the single Harman’s factor test. The results of the created (principal axis factoring and extraction) suggest that there are 43 factors. The maximum covariance explained by one factor in this study is 40.763 percent, according to Harman’s one-factor test.

## Data Analysis and Results

### Measurement Model

**FIGURE 1:**
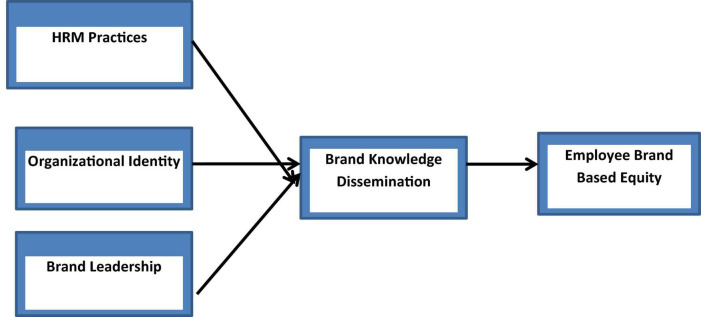
Conceptual model. HRM, human resource management; OI, organizational identity; Bl, brand leadership; EBBE, employee-based brand equity.

**FIGURE 2:**
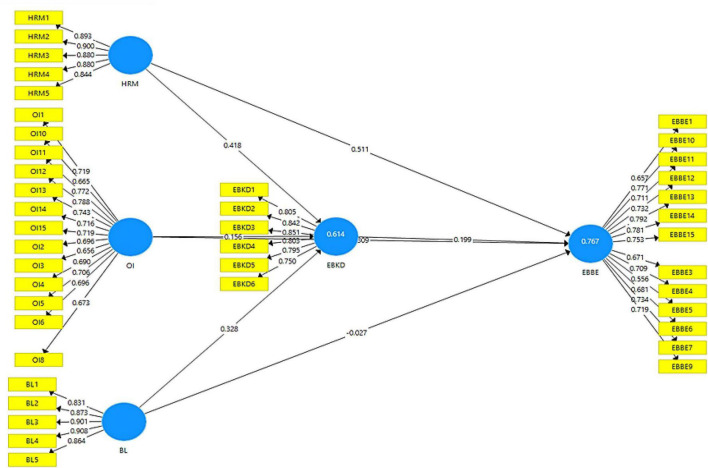
Output of measurement model algorithm. HRM, human resource management; OI, organizational identity; Bl, brand leadership; EBBE, employee-based brand equity.

The first step of PLS-SEM analysis is the assessment of measurement model (Figure 1). The output measurement model algorithm is shown in Figure 2. It explains the impact of independent variables on dependent variables.

In measurement model specification, examining the indicator reliability is considered the first step. As part of measurement model, a total of three items (EBBE2, OI7, and OI9) were removed due to low factor loading (<0.500). Reliability as assessed using Cronbach’s alpha and composite reliability: statistics for both were greater than the recommended value 0.700 (Wasko and Faraj, 2005). Convergent validity was acceptable because AVE was above 0.500 in majority.

Discriminant validity was assessed by comparison of the correlations among the latent variables and the square root of AVE (Fornell and Larcker, 1981) and heterotrait–monotrait ratio of correlation (Henseler et al., 2015), with values below the (conservative) threshold of 0.85. Hence, discriminant validity is established.

### Structural Model

**FIGURE 3:**
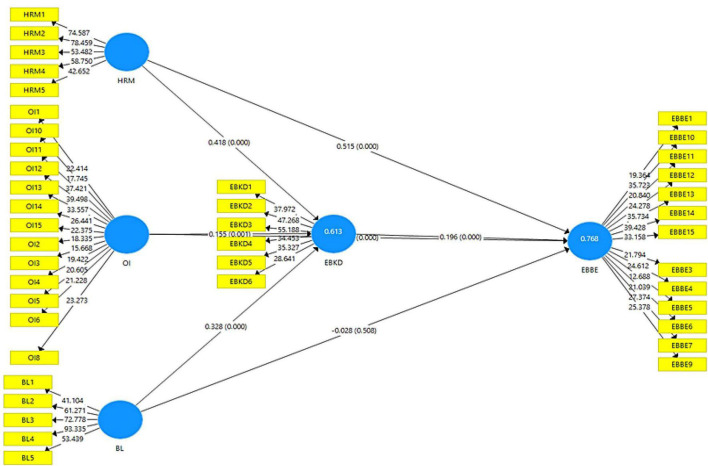
Structural model with moderation.

The structural model includes the paths hypothesized in the research framework (Figure 3). A structural model is assessed on the basis of *R*^2^, *Q*^2^, and significance of paths. *R*-square indicates the variance that has been described in dependent variable (Hair et al., 2014). Furthermore, the value of *R*^2^ can range from 0 to 1. Table 1 shows the value of 0.614 for employee brand knowledge dissemination and 0.767 for employee brand-based equity. As values of *R*-square are between 0 and 1, predictive capability is established. Moreover, to assess the goodness of model, hypotheses were tested to determine the significance of relationships.

**TABLE 1:** Discriminant validity (HTMT Ratio).

Fornell–Larcker criterion						Heterotrait–monotrait (HTMT) ratios					
	BL	EBBE	EBKD	HRM	OI		BL	EBBE	EBKD	HRM	OI
BL	0.876					BL					
EBBE	0.679	0.715				EBBE	0.711				
EBKD	0.644	0.695	0.808			EBKD	0.696	0.814			
HRM	0.598	0.650	0.722	0.879		HRM	0.639	0.792	0.792		
OI	0.519	0.617	0.573	0.591	0.712	OI	0.534	0.747	0.604	0.606	

*HRM, human resource management; EBBE, employee brand-based equity; OI, organizational identity; BL, brand leadership; EBKD, employee brand knowledge dissemination.*

H1 evaluates the relationship between HRM practices and employee brand-based equity (Tables 2–5). The results revealed that HRM practices have a significant impact on employee brand-based equity (β 0.515, *t* = 12.997, *p* = 0.000). Hence, H1 is supported. H2 examines the relationship between organizational identity and employee brand-based equity. The results showed that organizational identity positively influences the EBBE (β 0.310, *t* = 8.558, *p* = 0.000). Therefore, H2 is accepted. Moreover, H3 evaluates the relationship between brand leadership and employee brand-based equity. The results showed that brand leadership positively influences the employee brand-based equity (β = –0.028, *t* = 0.662, *p* = 0.508). Therefore, H3 is rejected.

**TABLE 2:** Direct effects.

Structural paths	Path coefficient (*t*-value)	Confidence interval	F2	*P*-values	Results
HRM - > EBBE	0.515 (12.997)	{0.516 to 0.655}	0.665	0.000	H1, Supported
OI - > EBBE	0.310 (8.588)	{0.260 to 0.419}	0.419	0.000	*H2, Supported*
BL - > EBBE	–0.028 (0.662)	{–0.411 to 0.125}	0.125	0.508	H3, Not-supported

*HRM, human resource management; EBBE, employee brand-based equity; OI, organizational identity; BL, brand leadership, level of significance = 0.05.*

**TABLE 3:** Demographic details.

Demographics	Frequency	Percent
**Gender**		
Male	270	
Female	151	
**Age (Years)**		
20–30	122	29%
30–40	181	43%
40–50	84	20%
>50	34	8%
**Organizational tenure**		
<1 Year	194	46.20%
1–3 Years	171	40.63%
4–6 Years	33	8%
>6 Years	23	6%

**TABLE 4:** Common method bias.

Factor	Initial eigenvalues			Extraction sums of squared loadings		
	Total	% of Variance	Cumulative %	Total	% of Variance	Cumulative %
1	18.097	42.085	42.085	17.528	40.763	40.763
2	3.712	8.634	50.719			
3	2.054	4.777	55.496			
4	1.245	2.895	58.391			
5	1.204	2.799	61.190			
6	1.164	2.707	63.897			
7	0.941	2.188	66.086			
8	0.914	2.125	68.210			
9	0.898	2.089	70.299			
10	0.801	1.863	72.162			
11	0.760	1.769	73.931			
12	0.685	1.594	75.525			
13	0.626	1.456	76.981			
14	0.589	1.371	78.351			
15	0.546	1.270	79.621			
16	0.519	1.206	80.828			
17	0.504	1.172	82.000			
18	0.495	1.152	83.152			
19	0.487	1.133	84.284			
20	0.454	1.055	85.339			
21	0.439	1.020	86.359			
22	0.411	0.957	87.316			
23	0.393	0.913	88.229			
24	0.383	0.892	89.121			
25	0.364	0.847	89.968			
26	0.346	0.805	90.773			
27	0.338	0.785	91.558			
28	0.334	0.776	92.335			
29	0.294	0.685	93.019			
30	0.291	0.677	93.696			
31	0.282	0.655	94.351			
32	0.264	0.615	94.966			
33	0.249	0.578	95.544			
34	0.243	0.565	96.109			
35	0.232	0.539	96.649			
36	0.221	0.514	97.162			
37	0.209	0.485	97.647			
38	0.200	0.464	98.112			
39	0.188	0.437	98.549			
40	0.169	0.393	98.942			
41	0.162	0.376	99.318			
42	0.153	0.357	99.675			
43	0.140	0.325	100.000			

**TABLE 5:** Factor loadings, Cronbach alpha, composite reliability, and AVE.

Construct	Items	Loadings	CR	Alpha	AVE	VIF
**Brand leadership**			0.943	0.924	0.767	
	BL1	0.831				2.222
	BL2	0.873				2.836
	BL3	0.901				3.466
	BL4	0.908				3.695
	BL5	0.864				2.794
**Employee brand-based equity**			0.931	0.920	0.512	
	EBBE1	0.657				1.691
	EBBE10	0.771				2.390
	EBBE11	0.711				1.931
	EBBE12	0.732				2.168
	EBBE13	0.792				2.410
	EBBE14	0.781				2.789
	EBBE15	0.753				2.487
	EBBE3	0.671				2.093
	EBBE4	0.709				2.143
	EBBE5	0.556				1.488
	EBBE6	0.681				1.897
	EBBE7	0.734				2.301
	EBBE9	0.719				1.948
**Brand knowledge dissemination**			0.919	0.894	0.654	
	EBKD1	0.805				2.018
	EBKD2	0.842				2.557
	EBKD3	0.851				2.599
	EBKD4	0.803				2.085
	EBKD5	0.795				1.995
	EBKD6	0.750				1.787
**HRM practices**			0.945	0.927	0.773	
	HRM1	0.893				3.599
	HRM2	0.900				3.818
	HRM3	0.880				2.936
	HRM4	0.880				2.880
	HRM5	0.844				2.387
**Organizational identity**			0.930	0.920	0.507	
	OI1	0.719				2.587
	OI10	0.665				1.823
	OI11	0.772				2.450
	OI12	0.788				2.621
	OI13	0.743				2.056
	OI14	0.716				1.948
	OI15	0.719				1.840
	OI2	0.696				2.743
	OI3	0.656				2.245
	OI4	0.690				2.224
	OI5	0.706				2.168
	OI6	0.696				2.029
	OI8	0.673				1.792

*HRM, human resource management; EBBE, employee brand-based equity; OI, organizational identity; BL, brand leadership; EBKD, employee brand knowledge dissemination.*

In order to test the significance of path coefficients, bootstrapping procedure was used as recommended by Sarstedt et al. (2017). Nevertheless, specific guidelines proposed by Preacher and Hayes (2008) were also considered for mediation analysis. Furthermore, 5,000 bootstrap samples were considered as suggested by Streukens and Leroi-Werelds (2016). This study’s bootstrap re-sampling 5000 also shows 95% confidence interval as shown in Table 2. A confidence interval different from zero indicates a significant relationship.

#### Mediation Analysis

Mediation analysis was performed to assess the mediating effect of brand knowledge dissemination. This study adopts variance accounted for VAF approach for mediation analysis (An et al., 2021). The results revealed that brand knowledge dissemination partially mediated the relationship between HRM practices and EBBE as value of VIF > 25. Similarly, the EBKD partially mediates the relationship of OI and EBBE as value of VAF > 25. EBKD fully mediates the relationship of BL and EBBE as value of VAF > 80 (see Table 6).

**TABLE 6:** Indirect effects.

Structural paths	Direct effect (*t*-value)	Indirect effect (*t*-value)	Total effect (*t*-value)	VAF	Interpretation	Results
HRM - > EBKD - > EBBE	0.515 (12.997)	0.180 (3.826)	0.695 (16.222)	26	Partial mediation	H4, Supported
OI - > EBKD - > EBBE	0.310 (8.588)	0.254 (2.798)	0.564 (8.972)	45	Partial mediation	H5, Supported
BL - > EBKD - > EBBE	0.028 (0.662)	0.0350(3.621)	0.322 (0.821)	89	Full mediation	H6, Supported

*HRM, human resource management; EBBE, employee brand-based equity; OI, organizational identity; BL, brand leadership, level of significance = 0.05.*

## Discussion

This research was conducted to evaluate the firm-level challenges of internationalization in Chinese context. China is the leading country after the United States investing single-handedly in international trading. Therefore, it was necessary to find out the right practices which could lead to overcoming the issues of organizations working internationally. Certain direct and indirect relationships of factors contributing toward EBBE were studied in this research. The first hypothesis was about the relationship of HRM practices with EBBE. The hypothesis got accepted due to the fact that if proper human resource practices are followed in any organization, then it leads to the development of brand equity, and in case of employees of the respective organization getting proper human resources, then it leads to EBBE. Similar kind of results was also reported previously (Aurand et al., 2005).

In these circumstances, HRM is the most significant and sensitive of all management areas in the local and international environment (Zhang, 2022). The other direct relationship of organizational identity also proved that if there is a clear identification of brands among the employees, then it could also lead to the development of strong EBBE at the organizational level. Organizational identity is a psychological paradigm that refers to what employees consider to be fundamental, distinctive, and long-lasting about their company (Albert and Whetten, 1985). Transforming a firm’s identity in internationalization scenario necessitates modifications to organizational members’ internal psychological structures and attitudes, which can lead to resistance and the inability to execute change (Nag et al., 2007). Similar kind of results was reported by Liu et al. (2020), indicating that brand identity among employees directs the positive outcomes of EBBE.

They also found that if proper brand identity is prevalent among employees, then it has an influence on quality of physical facility which ultimately affects consumer-based brand equity as well. The other direct effects of brand leadership were also studied in this research which were not significant in developing EBBE among employees. The possible reason of such outcome lies in the fact that leadership is sometimes not directly related to working employees of lower cadre. This is due to the competition sense among the employees and leaders. Although some of the previous studies proved that brand leadership could lead to the development of employee-based brand citizenship behavior (Shaari et al., 2015), they could not develop an analogy between brand leadership and EBBE. In some of the cases, brand leadership provided significant roles in developing consumer-based brand equity.

Consumers’ sense of a brand’s relative popularity as represented by brand knowledge and consumption is what popularity refers to. Consumers also wanted brand leadership to have a clear vision and to stay connected (Akbar et al., 2021; Khamwon and Sorataworn, 2021). The indirect and mediating role of brand knowledge dissemination proved its significance while mediating between HRM, organizational identity, brand leadership, and EBBE. All these hypotheses were accepted showing that if brand knowledge dissemination prevails in an organization, then it leads to enhanced EBBE. The direct relationship of brand leadership with EBBE was not significant, but mediation of brand knowledge dissemination provided anchorage in this relationship, indicating that with the help of brand knowledge dissemination from leadership to employees, it leads to the development of EBBE.

Employer branding contributes to employee behaviors and attitudes by delivering relevant and meaningful brand information. Furthermore, such psychological and cognitive shifts mean that workers transmit the brand value to customers as planned (Balemba Kanyurhi, 2016). Seemingly, it is critical to disseminate brand knowledge inside the corporate branding. Workers, for instance, are unable to convey an organization’s brand identity to consumers lacking brand-related knowledge. Moreover, few stated that “workers may feel directionless, grappling with understanding where, when, or to whom to devote their energy” in the lack of brand awareness (Zulkepli et al., 2015).

### Managerial Contributions

Employee-based brand equity facilitates the employee to develop a sense of comparison between perceived cost and benefits which affect them in future. There is certain benefit for developing EBBE among the employees as this study provides valuable insights that organizations develop EBBE among their employee through the brand knowledge dissemination. Brand knowledge dissemination among the employees strengthens the EBBE which provide attractive package for employee for their services. Similarly, management should take valuable insights from this study, improve their HRM practices, and develop organizational identity and brand-oriented leadership which helps to strengthen the EBBE among the employees. Rigorous training and promotions are the effective ways for brand knowledge dissemination among the employees and other stakeholders, which ultimately leads to strengthening the EBBE.

### Theoretical Contributions

This study contributes in the body of the literature in an effective way. EBKD mediates the relationship among HRM practices, organization identity, and brand leadership on employee brand-based equity which shows the importance of HRM practices, organizational identity, and brand leadership for strengthening the brand knowledge dissemination, which leads to EBBE. This study also theoretically contributes by finding that the brand knowledge dissemination among the employees fosters the employee brand-based equity which ultimately helps in reinforcing their organizational brand equity. This study provides valuable insights which help the research scholars and practitioners to further extend this area of study.

### Limitations and Future Recommendations

Although several important significances are found, this study has some limitations. First, time horizon of this study was cross sectional as data were collected at once so in future studies should adopt longitudinal strategy for data collection. Second, this study adopts convenience sampling for data collection which may raise the issue for generalization of study, so future studies should adopt any rigorous sampling technique for data collection. Using self-reporting measures may also cause issue of biasness, polarity, and other motives among such measures. In order to subjugate these limitations, future research can opt for qualitative approach using interviews or can implement triangulation techniques for behavioral observations. Future studies can add some more constructs for the completion of study. Future studies can obtain data from multiple sectors to assess the generalizability of the model. Moreover, brand knowledge management can be added in the model as mediator or moderator.

## Conclusion

One belt, one road initiative has been implemented during the second decade of 2000. The core objective of this initiative is economic integration, increasing China’s commercial viability, and creating opportunities for local businesses to become internalized. China’s ambition is to develop the world’s biggest campaign to showcase business and financial links with the entire world under its auspices. However, Chinese firms and businesses may be exposed to several challenges of internalization. In this scenario, effective HRM is playing a vital role in addressing the local and international issues faced by the organization. The novel contribution of this current research is to explore the internalization challenges faced at the organizational level due to the one belt, one road initiative. Drawing on the resource-based view theory, this study holds the view that effective HRM practices, organizational identity, and brand leadership are the strategic resources for any organization which helps them to create employee brand-based equity. This study also explores the indirect effect of brand knowledge dissemination among the HRM practices practice, organizational identity, and brand leadership on employee brand-based equity. The results of this study demonstrate that HRM practices and organizational identity have a direct influence on employee brand-based equity. However, the direct impact of brand leadership on the employee brand-based equity was found insignificant. Moreover, the results reveal that brand knowledge dissemination mediates the relationship between brand leadership and employee brand-based equity which depicts that brand leadership indirectly impacts brand-based equity through brand knowledge dissemination. Brand knowledge dissemination also mediates the relationship between HRM practice and organizational identity on employee brand-based equity. It is suggested from the finding of this study that organization management and policymakers should develop a rigorous strategy to ensure effective HRM practices. Organizational identity develops among the employees through conducting seminars and training. Knowledge management practices should be implemented in the organization to foster brand knowledge dissemination in the organizational environment.

## Data Availability Statement

The original contributions presented in the study are included in the article/supplementary material, further inquiries can be directed to the corresponding author/s.

## Author Contributions

XW was conceived and designed the concept, collected the data, wrote the manuscript, and read and agreed to the published version of the manuscript.

## Conflict of Interest

The author declares that the research was conducted in the absence of any commercial or financial relationships that could be construed as a potential conflict of interest.

## Publisher’s Note

All claims expressed in this article are solely those of the authors and do not necessarily represent those of their affiliated organizations, or those of the publisher, the editors and the reviewers. Any product that may be evaluated in this article, or claim that may be made by its manufacturer, is not guaranteed or endorsed by the publisher.
